# Green, Black and Rooibos Tea Inhibit Prostaglandin E2 Formation in Human Monocytes by Inhibiting Expression of Enzymes in the Prostaglandin E2 Pathway

**DOI:** 10.3390/molecules27020397

**Published:** 2022-01-08

**Authors:** Alexander Hedbrant, Ingrid Persson, Ann Erlandsson, Jonny Wijkander

**Affiliations:** 1 Department of Health Sciences, Karlstad University, SE-651 88 Karlstad, Sweden; alexander.hedbrant@oru.se (A.H.); annalenasjobeck@gmail.com (I.P.); ann.erlandsson@kau.se (A.E.); 2 School of Medical Sciences, Faculty of Medicine and Health, Örebro University, SE-701 82 Örebro, Sweden; 3 Department of Environmental and Life Sciences/Biology, Karlstad University, SE-651 88 Karlstad, Sweden

**Keywords:** green tea, black tea, rooibos tea, epigallocatechin gallate, quercetin, monocytes, microsomal prostaglandin E2 synthase, cyclooxygenase, prostaglandin E2, phospholipase A2

## Abstract

The formation of prostaglandin E2 (PGE2) is associated with adverse inflammatory effects. However, long-term treatment with nonsteroidal anti-inflammatory drugs (NSAIDs) comes with risk of severe side effects. Therefore, alternative ways to inhibit PGE2 are warranted. We have investigated the effects of tea extracts and the polyphenols epigallocatechin gallate (EGCG) and quercetin on PGE2 formation, determined by immunoassay, and protein expression, determined by immunoblotting, of cytosolic phospholipase A2 (cPLA2), cyclooxygenase 2 (COX-2) and microsomal PGE synthase-1 (mPGES-1) in human monocytes. Green and black tea extracts, and with a lower potency, Rooibos tea extract, inhibited lipopolysaccharide (LPS) and calcium ionophore-induced PGE2 formation. In addition, all tea extracts inhibited the LPS-induced expression of mPGES-1, and the green and black tea extracts also inhibited, to a lesser extent, COX-2 expression. The tea extracts only marginally reduced cPLA2 expression and had no effect on COX-1 expression. EGCG, present in green and black tea, and quercetin, present in all three teas, also inhibited PGE2 formation and expression of mPGES-1, COX-2 and cPLA2. Cell-based and cell-free assays were also performed to evaluate direct effects on the enzymatic activity of COX and PGE synthases. Mainly, the cell-free assay demonstrated partial inhibition by the tea extracts and polyphenols. However, the inhibition required higher doses compared to the effects demonstrated on protein expression. In conclusion, green and black tea, and to a lesser extent Rooibos tea, are potent inhibitors of PGE2 formation in human monocytes, and mediate their effects by inhibiting the expression of the enzymes responsible for PGE2 formation, especially mPGES-1.

## 1. Introduction

Consumption of tea has been attributed health beneficial effects, and it has been suggested to reduce the risk of, e.g., cardiovascular diseases and cancer [1,2]. Green tea and black tea from the leaves of *Camellia sinensis* are rich in polyphenols, including catechins and flavonols. The main catechin found in green tea is epigallocatechin gallate (EGCG), and it is a major constituent of the polyphenols, but epicatechin gallate, epigallocatechin and catechin gallate are also present [3,4]. EGCG is the most well-studied of the tea flavonoids, and it has been suggested to have beneficial effects in humans, predominantly due to its antioxidant properties [5,6]. Black tea also contains substantial amounts of polyphenols. However, its content of EGCG is lower than of green tea due to oxidation of the catechins, resulting in dimers and polymers of the catechins, named theaflavins and thearubigins [3]. Rooibos tea, which is produced from the leaves and stems of *Aspalathus linearis*, does not contain catechins but does contain flavonols such as quercetin, although in lower amounts than those found in green and black tea [3,7].

Prostanoids are signaling molecules which act in an autocrine or paracrine fashion to exert various functions, including pro- and anti-inflammatory effects, vascular toning, smooth muscle contraction, kidney function and induction of fever and pain [8]. Prostaglandin E2 (PGE2) is the principal prostaglandin produced during inflammation, and its formation is associated with adverse effects in various diseases, including cancer, atherosclerosis and chronic inflammatory or autoimmune diseases [9,10]. Nonsteroidal anti-inflammatory drugs (NSAIDs), which inhibit cyclooxygenase (COX) enzymes and thereby PGE2 formation, are one of the most abundantly used group of drugs worldwide. However, long-term use of NSAIDs as treatment or prophylaxis of diseases is problematic due to the risk of serious side effects, including gastrointestinal bleeding, via inhibition of prostanoid formation. Therefore, there is a need for alternative ways to inhibit PGE2 formation.

Prostanoids are derived from the fatty acid arachidonic acid, which is released from membrane phospholipids by the action of cytosolic phospholipase A2 (cPLA2). Free arachidonic acid is converted to PGH2 by the enzymes cyclooxygenase (COX)-1 or COX-2, which can subsequently be converted into the prostanoids PGI2, PGD2, PGF2α, PGE2 or thromboxane A2 by the action of their respective synthases [11]. There are three PGE2 synthases, namely microsomal PGE synthase (mPGES)-1, mPGES-2, and cytosolic PGES (cPGE2) [12]. mPGES-1 is inducible by inflammatory stimuli, whereas mPGES-2 and cPGES are constitutively expressed. The expression of cPLA2 and COX-2 can also be induced, while COX-1 is constitutively expressed by most cells.

In the present study, we investigated the inhibitory effects of green tea, black tea, Rooibos tea extracts, and the polyphenols EGCG and quercetin on the formation of PGE2 and the expression of the enzymes involved in its formation in human monocytes. We demonstrated that green and black tea, and to a lesser extent Rooibos tea, inhibited PGE2 formation in human monocytes. In addition, we found that the tea extracts inhibited the LPS-induced protein expression of enzymes in the PGE2 synthesis pathway, primarily mPGES-1. Similar results were observed for EGCG and quercetin, and the results suggest EGCG to be the primary component in green tea mediating the observed effects.

## 2. Results

### 2.1. Tea Extracts, EGCG, and Quercetin Inhibits LPS- and Calcium Ionophore-Induced PGE2 Formation in Human Monocytes

**Figure 1:**
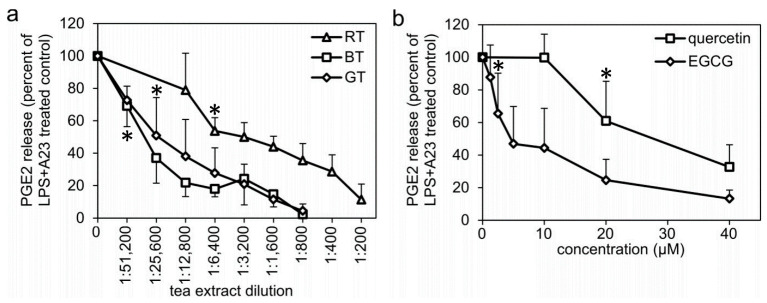
Effect of treatment with (**a**) tea extracts, or (**b**) polyphenols on PGE2 formation in human monocytes. The monocytes were pretreated with LPS for 24 h (together with indicated tea/polyphenol treatment) prior to washing and addition of fresh culture media and stimulation with 4 µM A23187 for 45 min. The culture media was collected and its PGE2 content was analyzed with an enzyme immunoassay. Results for each individual data point represent mean value ± SD from 3 to 9 independent experiments from different preparations of human monocytes, expressed as percent of the amount PGE2 formed in the LPS + A23187-treated control monocytes. BT: black tea extract, GT: green tea extract, RT: Rooibos tea extract, EGCG: epigallocatechin gallate. * denotes the highest dilution/lowest concentration mediating a significant inhibition (*p* < 0.05).

Human monocytes were pretreated with LPS for 24 h prior to assessment of the amount of PGE2 formed by the cells in response to a 45 min stimulation with the calcium ionophore A23187. Treatment with green, black, or Rooibos tea extracts during the 24 h LPS pretreatment dose-dependently inhibited the amount of PGE2 formed (Figure 1a). The green and black tea extracts were equally potent inhibitors, whereas the Rooibos tea extract was less potent. Treatment with EGCG or quercetin also inhibited PGE2 formation in a dose-dependent manner, with EGCG being more potent (IC_50_ value, approximately 5 µM) compared to quercetin (IC_50_ value, approximately 30 µM) (Figure 1b). Monocytes treated with the COX inhibitor indomethacin (10 µM), or monocytes either not pretreated with LPS or not stimulated with A23187, revealed very low amounts of PGE2: about 10% of the PGE2 formed in response to LPS + A23187 treatment (Supplementary Materials, Table S1).

### 2.2. Tea Extracts, EGCG, and Quercetin Inhibits the Protein Expression of Enzymes of the PGE2 Synthesis Pathway

**Figure 2:**
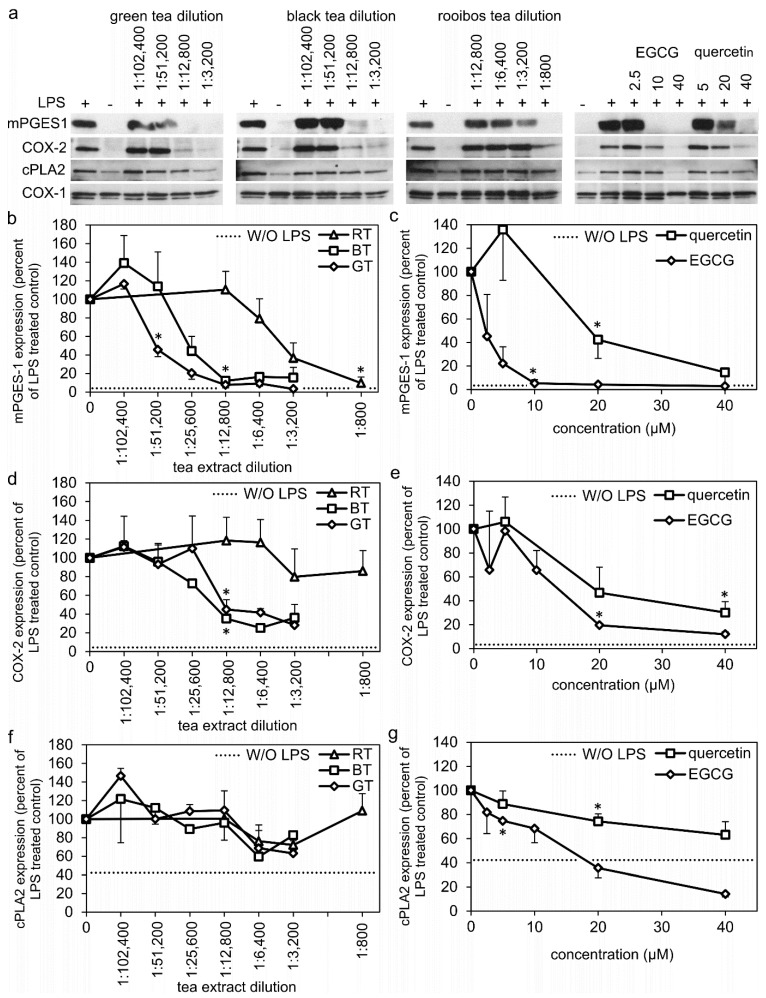
(**a**) Representative Western blots of mPGES-1, COX-1, COX-2, and cPLA2 expression in human monocytes after indicated treatments for 24 h. (**b**–**g**) Densitometric quantification of Western blot results compared to LPS treatment alone. COX-1 expression was used as the internal loading control. Results for each individual data point represent mean value ± SEM of 1–5 independent experiments with human monocytes from different preparations. Dotted lines (**b**–**g**) indicate the basal expression (without LPS treatment) of each protein. BT: black tea extract, GT: green tea extract, RT: Rooibos tea extract, EGCG: epigallocatechin gallate. * denotes the highest dilution/lowest concentration mediating a significant inhibition (*p* < 0.05). Uncropped versions of the Western blots are shown in Supplementary Materials, Figure S1.

The monocytes had no, or very low basal protein expression of mPGES-1 and COX-2. However, both were highly induced by LPS treatment (Figure 2). COX-1 was unaffected by LPS (LPS treatment resulted in 89 ± 17% of nontreated monocytes (*n* = 15). Nontreated monocytes had a basal protein expression of cPLA2, but LPS treatment increased the expression about 2-fold (Figure 2). Treatment of the monocytes with green, black, or Rooibos tea extracts all inhibited the LPS-induced protein expression of mPGES-1 in a dose-dependent manner with a potency of green tea > black tea > Rooibos tea (Figure 2a,b). The LPS-induced COX-2 expression was dose-dependently inhibited by treatment with the green and black tea extracts, at a similar potency, while no significant inhibition was observed by the Rooibos tea extract (Figure 2a,d). The green and black tea extracts inhibited the protein expression of mPGES-1 with a greater potency and efficacy compared to their inhibition of COX-2 (compare 3b,d). cPLA2 expression was not significantly inhibited by any of the tested tea extracts (Figure 2f). Neither tea extracts, EGCG or quercetin had any effect on the COX-1 expression in monocytes (Figure 2a).

Treatment with EGCG or quercetin dose-dependently inhibited the LPS-induced protein expression of mPGES-1 and COX-2, with EGCG being a more potent inhibitor than quercetin, especially for mPGS-1 (Figure 2a,c,e). Treatment with EGCG and quercetin also inhibited cPLA2 protein expression dose-dependently but to a different extent. EGCG inhibited cPLA2 to levels below the basal expression at concentrations of 20–40 µM, while quercetin marginally inhibited cPLA2 expression at the same doses (Figure 2a,g).

### 2.3. Effects of Treatment with Tea Extracts, EGCG, and Quercetin on the Enzymatic Activity of Proteins of the PGE2 Synthesis Pathway

**Figure 3:**
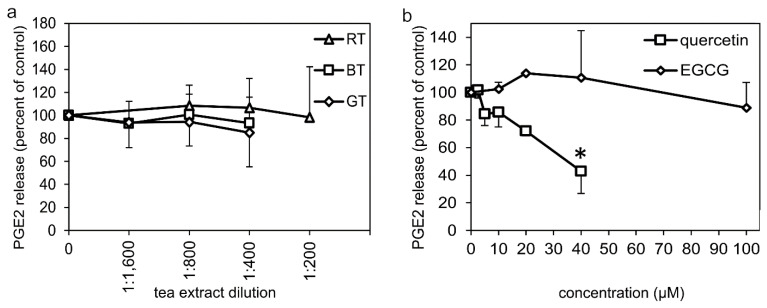
Cellular assay for the combined enzymatic activity of cPLA2, COX and PGE synthases to produce PGE2 in LPS-pretreated (24 h) monocytes during a 1 h treatment with (**a**) tea extracts; (**b**) EGCG or quercetin. PGE2 formation was initiated by stimulation with 4 µM A23187 during the last 45 min of the 1 h treatment with tea extracts or polyphenols. Results for each data point represent the mean value ± SD of 2–7 independent experiments from different monocyte preparations, and are expressed as percent of PGE2 released from the LPS + A23187-treated controls. * denotes a significant inhibition (*p* < 0.05). BT: black tea extract, GT: green tea extract, RT: Rooibos tea extract, EGCG: epigallocatechin gallate.

Having established the effects of the tea extracts and polyphenols on the protein expression of the enzymes involved in formation of PGE2, we further wanted to investigate if the extracts and polyphenols had any effects on the activity of the enzymes. A cellular assay to determine effects on the combined enzymatic activity of enzymes in the PGE2 synthesis pathway was performed using LPS-pretreated monocytes and subsequent treatment with tea extracts, EGCG, or quercetin for 1 h, together with A23187 stimulation. Treatment with quercetin dose-dependently inhibited PGE2 formation, while no inhibitory effect was observed with EGCG or any of the tea extracts (Figure 3a,b). For comparison, treatment with indomethacin (10 µM) resulted in complete inhibition (100 ± 1% *n* = 4, *p* < 0.05) of the LPS + A23187-stimulated PGE2 formation.

**Figure 4:**
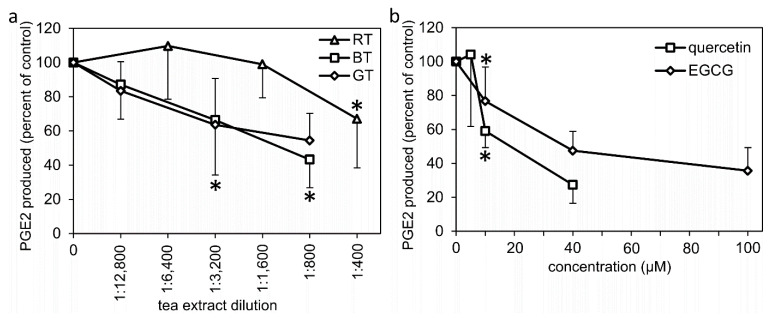
Cell-free assay for the combined enzymatic activity of COX and PGES to convert arachidonic acid into PGE2. A 1700× *g* cell lysate supernatant from the LPS-treated monocytes was used as the enzyme source and preincubated for 15 min with (**a**) tea extracts; (**b**) EGCG or quercetin. The reaction was initiated by addition of 0.5 µM arachidonic acid and was allowed to proceed for 30 min at 37 °C, followed by determination of PGE2 formation with an enzyme immunoassay. Results for each individual data point represent the mean value ± SD of 5–6 independent experiments and are expressed as percent of vehicle-treated control (PBS or DMSO). * denotes the highest dilution/lowest concentration mediating a significant inhibition (*p* < 0.05). BT: black tea extract, GT: green tea extract, RT: Rooibos tea extract, EGCG: epigallocatechin gallate.

In addition to the cellular assay, a cell-free assay for the direct effects of tea extracts, EGCG, or quercetin on the combined enzymatic activity of COX and PGE synthases was also performed. Green and black tea extracts inhibited PGE2 formation in a dose-dependent manner, with similar potency, while the Rooibos tea extract was less potent (Figure 4a). Quercetin and EGCG also dose-dependently inhibited PGE2 formation down to 50% of the control, at approximately 20 µM and 40 µM, respectively (Figure 4b). For comparison, indomethacin (10 µM) inhibited PGE2 formation down to 26 ± 15% of the control (*n* = 6, *p* < 0.05).

## 3. Discussion

PGE2 is an important and potentially harmful inflammatory mediator, and the evidence supports cPLA2 as the main enzyme in mobilizing arachidonic acid during inflammation, and the inducible enzymes COX-2 and mPGES-1 for the formation of PGE2 [13]. PGE2 formation also takes place under physiological (noninflammatory) conditions, and here the constitutively expressed COX-1 and cPGES are the main enzymes involved [12,14]. We investigated the effects of tea extracts and polyphenols on PGE2 formation, as well as their effects on the protein expression of the involved enzymes in human monocytes. Our results demonstrate that extracts of green, black, and Rooibos tea inhibited the LPS-induced protein expression of mPGES-1 and COX-2 in a dose-dependent manner, while they only marginally affected the expression of cPLA2 and had no effect on the expression of COX-1. Both green and black tea extracts strongly inhibited mPGES-1 and COX-2 expression at dilutions of 12,800 and lower, resulting in near complete inhibition of mPGES-1 and about 70% inhibition of COX-2. Partial inhibition of mPGES-1 was observed at even higher dilutions, especially for the green tea extract, with significant inhibition at 1:51,200 dilution. The Rooibos tea extract mainly affected mPGES-1 expression, but with a much lower potency compared to the green and black tea extracts. To our knowledge, no studies have been published regarding the effects of tea extracts on the protein expression of mPGES-1. However, both green and black tea have been shown to inhibit COX-2 expression in lung cancer cell lines [15] and in vivo in rats [16].

Regarding the LPS-induced signal transduction pathways for transcriptional activation of the genes for COX-2 and mPGES-1, both coordinated [17] and uncoupled [18] regulation of the enzymes have been demonstrated. In addition, studies have shown a difference in the transcriptional kinetics, with COX-2 being transcribed at earlier time-points than mPGES-1 following LPS treatment [18,19]. Our results, showing that green tea extract was somewhat more potent in inhibiting the LPS-induced protein expression of mPGES-1 than that of COX-2, were consistent with the possibility of different signaling pathways for the regulation of the expression of the two enzymes.

The effects of the tea extracts on the expression of mPGES-1 and COX-2 were accompanied by inhibition of PGE2 formation. Green and black tea extracts have previously been shown to inhibit PGE2 formation in human monocytes [20], but only at dilutions of 1:1700 or less. This is a considerably higher dose than the 1:25,600 dilution that mediated a significant inhibition in the current study. Comparing the results for the inhibition of PGE2 formation with the inhibition of the studied enzymes, only mPGES-1 expression was strongly and significantly inhibited by the 1:25,600 dose of the green tea extract, and a similar result was observed for black tea. The results for the Rooibos tea extract also suggest that inhibition of mPGES-1 expression and PGE2 formation coincide at similar doses, although at a lower dilution compared to green and black tea. Taken together, these results suggest that the inhibitory effect of the tea extracts on mPGES-1 protein expression has a direct impact on the amount of PGE2 formed.

Catechins are a group of polyphenols which are found in substantial amounts in green and black tea, but not in Rooibos tea [3,4]. In addition to catechins, all three teas contain flavonols, of which quercetin dominates [21]. Both the catechin EGCG and the flavonol quercetin dose-dependently inhibited the LPS-induced protein expression of mPGES-1, COX-2, and cPLA2 as well as PGE2 formation in monocytes. In accordance with our results, EGCG has been shown to inhibit LPS-induced PGE2 formation in human whole blood and in mouse osteoblasts [22,23]. The content of the four main green tea catechins—epicatechin, epigallocatechin, epicatechin gallate, and EGCG—in tea extracts from the same tea products and extraction method, as used in our study, have previously been reported [4]. Accordingly, our green tea extract at a 12,800 dilution should contain about 2.5 µM EGCG. Thus, our results indicate that the EGCG content of green tea, to a large extent, is responsible for the inhibition of the expression of mPGES-1 and COX-2 as well as for PGE2 formation. Regarding black tea, the content of EGCG was, in the same study [4], reported to be about seven times lower compared to the green tea extract, and other studies confirm similar or even lower values [3]. In the current study, black tea extract was found to inhibit PGE2 formation and the expression of the enzymes at doses comparable to those of the green tea extract. This suggests that compounds besides EGCG, to a large extent, are responsible for the effects of black tea. EGCG has been shown to inhibit COX-2 expression in THP-1 monocytes [24] and other cancer cell lines [25,26,27]. To our knowledge, only one study on the effect of EGCG on mPGES-1 expression has been published. In mouse osteoblasts, Tominari et al. [23] showed a partial inhibition (about 40% inhibition) of LPS-induced mPGES-1 mRNA expression by 30 µM EGCG. This could be compared to the complete inhibition of the protein expression of mPGES-1 that we observed in human monocytes in response to 10 µM EGCG.

The direct effects of tea extracts, EGCG, and quercetin on the activity of the enzymes involved in PGE2 formation was investigated using two experimental approaches: a cell-based assay, where the tea extracts or polyphenols were added 24 h after LPS pretreatment but prior to A23187 stimulation, and a cell-free assay of combined COX and PGES activity. Of the studied compounds and tea extracts, only quercetin showed a dose-dependent inhibition of PGE2 formation in both assays. The tea extracts and EGCG inhibited PGE2 formation in the cell-free assay for combined COX and PGES enzymatic activity, but did not inhibit PGE2 formation in the cell-based assay. EGCG has previously been shown to inhibit the activity of mPGES-1 but not COX-2 in cell-free assays [22]. The discrepancy of the results for the cell-based and cell-free assays for EGCG could be due to the difficulty of EGCG to pass cell membranes. Previous studies have shown EGCG and other tea catechins to bind to and interact with lipid bilayers, thus preventing EGCG from reaching the cytosol [28,29]. Quercetin, on the other hand, has been shown to pass cell membranes [30], likely explaining the similar effects in our cell-based and cell-free assays for this compound. Although some direct effects on the enzymatic activities of COX and PGES enzymes were observed, especially in the cell-free model, higher doses were required for these effects compared to the inhibition observed on the protein expression of the enzymes.

In humans, plasma levels of EGCG have been shown to reach concentrations of about 1 µM in response to a single dose of EGCG [31,32] or green tea extracts [33]. Our results on the inhibitory effects on PGE2 formation and mPGES-1 expression in response to green and black tea extracts at dilutions from 1:25,600 and for EGCG at 2.5 µM could therefore be considered physiologically relevant concentrations.

Although other studies have shown the inhibition of COX-2 protein expression and PGE2 formation in response to tea extracts, EGCG, and quercetin, our study is the first to compare extracts from green, black, and Rooibos tea on PGE2 formation and protein expression of mPGES-1. Our results demonstrate that green and black tea extracts are highly potent inhibitors of PGE2 formation in human monocytes, and that they to a large extent mediate their effects via the inhibition of the expression of mPGES-1. EGCG is likely the main polyphenol in green tea responsible for the inhibition of PGE2 formation and mPGES-1 expression, while in black tea mainly other polyphenols are responsible for the inhibition.

## 4. Materials and Methods

### 4.1. Polyphenols and Tea Extracts

EGCG and quercetin (both from Sigma Aldrich, St. Louis, MO, USA) were dissolved in DMSO and stored in aliquots at −20 °C until use. Green tea (Japanese Sencha, imported by Charabang, Stockholm, Sweden), black tea (Indian Assam B.O.P., imported by Norrköpings Kolonial, Norrköping, Sweden), and Rooibos tea (imported by Norrköpings Kolonial, Norrköping, Sweden) were prepared by adding 20 mL of boiling PBS to 1 g of tea, generating 1:20 dilutions of the tea extracts. After 5 min incubation for the green and black teas, or 10 min for the Rooibos tea, the extracts were filtered twice through a 0.22 µm sterile filter and stored in aliquots at −20 °C until use. Extracts from the same tea brands prepared using the same extraction methods have previously been characterized, and 1:20 dilutions of green and black tea extracts contained 1.6 mM and 0.22 mM of EGCG, respectively, while Rooibos tea extract did not contain any EGCG [4].

### 4.2. Isolation and Culture of Human Monocytes

Buffy coats were obtained from the division of Clinical and Immunological Transfusion Medicine, Uppsala University Hospital (Uppsala, Sweden) from healthy blood donors. For 50 mL of buffy coat, an equal volume of PBS with 3 mM EDTA (PBS/EDTA) was added, and peripheral blood mononuclear cells (PBMCs) were isolated and collected using gradient centrifugation on a Ficoll paque PLUS (GE Healthcare, Little Chalfont, UK) by centrifugation at 900× *g* for 30 min at room temperature. The separated PBMCs were washed 5 times with PBS/EDTA using repeated centrifugations for 10 min each, the first time at 500× *g*, and the following 4 washes at 200× *g*. The PBMCs were resuspended in 100 mL of RPMI 1640 (RPMI) (Life Technologies, Carlsbad, CA, USA) supplemented with 2 mM L-glutamine (Life Technologies), 100 U/mL penicillin and 100 µg/mL streptomycin (Life Technologies) (denoted RPMI) and seeded onto 24- or 6-well polystyrene cell culture plates or 58 cm^2^ cell culture dishes (Greiner Bio-One, Frickenhausen, Germany) by adding 0.4 mL, 2 mL or 14 mL cell suspension per well/dish, respectively. The monocytes were allowed to adhere for 2 h, followed by 3 washes with PBS to remove nonadherent PBMCs. The cells were then cultured for 24 h in RPMI with 5% fetal calf serum (FCS) (Thermo Scientific Inc., Waltham, MA, USA). Next, the monocytes were washed twice with PBS and RPMI without FCS was added before the start of treatments. The cells were kept in a humidified atmosphere at 37 °C with 5% CO_2_ during all incubation times.

### 4.3. PGE2 Formation in Human Monocytes

Two different experimental setups were performed for the analysis of the effects of tea extracts or polyphenols on the production of PGE2 in human monocytes. The monocytes were generated as described above (cultured on 24-well cell culture plates). To assess the gene regulatory effects of the tea extracts or polyphenols, the experimental setup included a 24 h pretreatment with tea extracts, EGCG or quercetin, together with 1 µg/mL lipopolysaccharide (LPS, Sigma Aldrich, St. Louis, MO, USA) followed by 2 washes with PBS, the addition of fresh media and stimulation with 4 µM A23187 (Sigma Aldrich) for 45 min and the collection of culture media (experimental setup A, Table 1). In a second experimental setup, effects of the tea extracts or polyphenols on the enzymatic activity was assessed. In this setup, LPS-pretreated cells (24 h) were washed with PBS, received fresh media, then treated with tea extracts, EGCG or quercetin for 15 min prior to stimulation with 4 µM A23187 for 45 min (experimental setup B, Table 1). EGCG and quercetin were dissolved in DMSO and added to culture dishes, resulting in a final DMSO concentration of 0.2%. Control dishes received an equivalent volume of DMSO. Control dishes for experiments with tea extracts received an equivalent volume of PBS. The collected culture media was stored at −20 °C until PGE2 analysis using an enzyme immunoassay (Cayman Chemical, Ann Arbor, MI, USA) according to the manufacturer’s instructions.

### 4.4. Immunoblotting

The monocytes were cultured as described above (Table 1, Setup A) on 6-well cell culture plates. Treatments started with the addition of tea extracts, EGCG or quercetin. After 30 min, 1 µg/mL LPS was added, and cells were incubated for a total of 24 h. Following treatment, the cells were lysed in 150 µL Laemmli’s sample buffer supplemented with protease inhibitors (20 µg/mL aprotinin, 10 µg/mL leupeptin, 5 mM phenylmethanesulfonyl fluoride (all from Sigma Aldrich), boiled for 5 min, and stored at −20 °C until analysis.

An equal volume of each cell lysate was subjected to SDS-PAGE (12% acrylamide gel), and the separated proteins were transferred onto a polyvinylidene fluoride membrane (Bio-Rad, Hercules, CA, USA). After blocking with an appropriate blocking buffer (TBS with 0.1% Tween-20 (TBS-T) supplemented with either 5% bovine serum albumin (BSA) for the mPGES-1 antibody or 3% gelatin for the cPLA2, COX-1 and COX-2 antibodies), the membranes were incubated with a primary antibody overnight at 4 °C. Primary antibodies included COX-1 (goat polyclonal, sc-1752) diluted 1:500 in TBS-T, COX-2 (goat polyclonal, sc-1745) diluted 1:500 in TBS-T, cPLA2 (goat polyclonal, sc-1724) diluted 1:300 in TBS-T or mPGES-1 (mouse monoclonal, sc-166308) diluted 1:250 in TBS-T supplemented with 5% BSA (all from Santa Cruz Biotechnology Inc., Dallas, TX, USA). An appropriate secondary antibody (antigoat secondary sc-2020 diluted 1:10,000 in TBS-T or antimouse secondary sc-2005 diluted 1:5000 in TBS-T, both from Santa Cruz Biotechnology Inc., Dallas, TX, USA) was applied for 1 h at room temperature and the signals were developed with the SuperSignal West Pico Chemiluminescent Substrate (Thermo Scientific Inc., Rochester, NY, USA) and visualized by exposure to Amersham Hyperfilm ECL (GE Healthcare). Following signal detection, the membranes were stripped for 25 min with the Restore Western Blot Stripping Buffer (Thermo Scientific Inc.) and reused for subsequent detection with another primary antibody, until all four proteins studied (i.e., COX-1, COX-2, mPGES-1 and cPLA2) had been detected for each membrane.

### 4.5. Preparation of Subcellular Fractions for Cell-Free Assay of Combined Enzymatic Activity of COX and PGES

Monocytes were cultured as described above on cell culture dishes, and treated with 1 µg/mL LPS for 24 h. Following treatment, the cells were washed twice with PBS and detached by scraping the cells from the dishes in buffer X (80 mM KCl, 10 mM HEPES, 1 mM EDTA, pH 7.4) supplemented with protease inhibitors (20 µg/mL aprotinin, 10 µg/mL leupeptin, 5 mM PMSF). The cells were lysed by sonication and the samples were centrifuged at 1700× *g* for 10 min at 4 °C. Tea extract, EGCG or quercetin was added to the 1700× *g* supernatant and diluted with buffer X supplemented with 2 mM L-glutathione (Sigma Aldrich), to a final reaction volume of 100 µL. Controls were treated with a vehicle (PBS or DMSO). Following a 15 min preincubation, the reaction was started by the addition of 0.5 µM arachidonic acid (Sigma Aldrich) and allowed to proceed for 30 min at 37 °C. The reaction was terminated by the addition of 400 µL ice-cold methanol. The methanol was dried under a gentle stream of N2, and the samples were redissolved in 300 µL RPMI media. The PGE2 content was analyzed with an enzyme immunoassay (Cayman Chemical) according to the manufacturer’s instructions.

### 4.6. Statistics

Results are expressed as mean value ± standard deviation (SD) or standard error of the mean (SEM). The one-sample t-test was used to test if significant inhibition regarding PGE2 formation or protein expression was achieved by the treatments with polyphenols or tea extracts, compared to the control samples (set to 100%).

## Figures and Tables

**Table 1:** Flowchart for the experimental setups used for the assessment of PGE2 formation in human monocytes when treated with tea extracts, EGCG or quercetin. The only difference between the two experimental setups is the indicated timing of treatment with tea extracts or polyphenols.

Treatment	Addition at Time	Setup A	Setup B
Tea extracts or polyphenols	0 h	X	-
LPS, 1 µg/mL	30 min	X	X
Wash, addition of fresh culture media	24 h	X	X
Tea extracts or polyphenols	24 h	-	X
A23187, 4 µM	24 h 15 min	X	X
Collection of culture media for PGE2 analysis	25 h	X	X
Collection of cell lysates for immunoblotting	25 h	X	-

## Data Availability

The data presented in this study are available on request from the corresponding author.

## Supplementary Materials

The following are available online, Table S1: Effects of indomethacin on the LPS + A23187-induced PGE2 release in human monocytes, Figure S1: Uncropped version of the Western blots presented in Figure 2.

## Sample Availability

Samples of the compounds are available from the authors.
