# Supplementary material for: Green, Black and Rooibos Tea Inhibit Prostaglandin E2 Formation in Human Monocytes by Inhibiting Expression of Enzymes in the Prostaglandin E2 Pathway
Source: Molecules. 2022 Jan 8;27(2):397. doi: 10.3390/molecules27020397 (PMC8778366; doi:10.3390/molecules27020397)
Supplement: Supplementary file 1 [file molecules-27-00397-s001.zip › molecules-1530080-supplementary.pdf]

Supplementary Materials

Table S1. Effects of indomethacin on the LPS+A23187 induced PGE2 release in human monocytes. The monocytes were pre-treated, or not pre-treated, with LPS for 24 h prior to wash and addition of fresh culture media and stimulation with 4 μM A23187 for 45 min. Indomethacin or DMSO (vehicle for indomethacin and A23187) was added 15 min before A23187. The culture media was collected and its PGE2 content was analyzed with an enzyme immunoassay. The P-values were calculated using the paired students t-test (n=4). LPS: Lipopolysaccharide, PGE2: prostaglandin E2, A23187; calcium ionophore A23187, SD: standard deviation.

Treatment	PGE2 (pg/ml) Mean values	SD (pg/ml)	% of LPS+A23187	P-value
LPS+DMSO+A23187	1339	572	100	
LPS+Indomethacin+A23187	124	33	9.2	0.021
LPS+DMSO+DMSO	134	45	10.0	0.021
DMSO+A23187	129	34	9.6	0.028

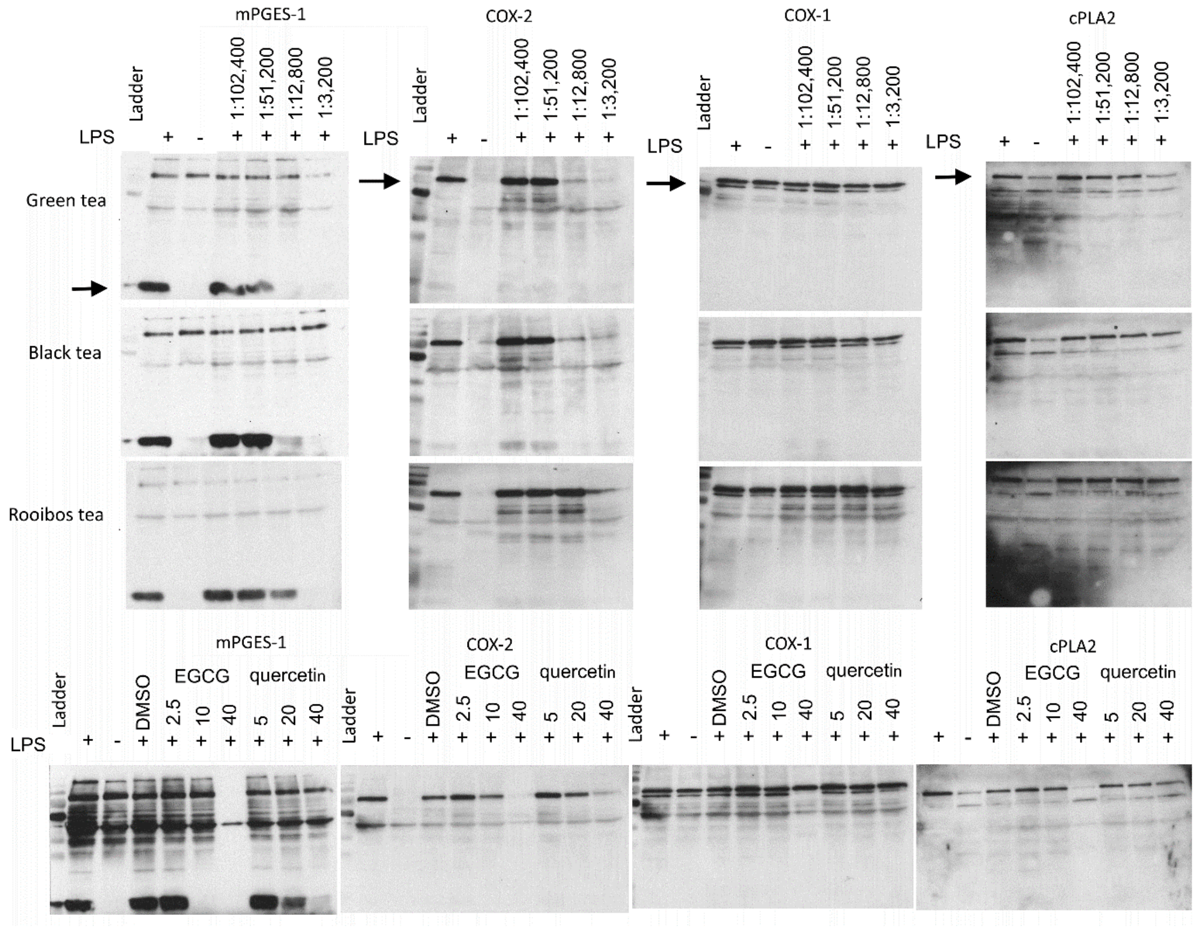

Figure S1. Un-cropped versions of the Western blots presented in Figure 2. LPS: lipopolysaccharide, DMSO: dimethylsulfoxide, EGCG: epigallecatechin gallate.
